# HOXA5 Is Recognized as a Prognostic-Related Biomarker and Promotes Glioma Progression Through Affecting Cell Cycle

**DOI:** 10.3389/fonc.2021.633430

**Published:** 2021-08-17

**Authors:** Fengqin Ding, Ping Chen, Pengfei Bie, Wenhua Piao, Quan Cheng

**Affiliations:** ^1^Department of Clinical Laboratory, People’s Hospital of Ningxia Hui Autonomous Region, First Affiliated Hospital of Northwest Minzu University, Yinchuan, China; ^2^Medical Experiment Center, General Hospital of Ningxia Medical University, Yinchuan, China; ^3^Department of Neurosurgery, People’s Hospital of Ningxia Hui Autonomous Region, First Affiliated Hospital of Northwest Minzu University, Yinchuan, China; ^4^Department of Neurosurgery, Xiangya Hospital, Central South University, Changsha, China; ^5^National Clinical Research Center for Geriatric Disorders, Xiangya Hospital, Central South University, Changsha, China; ^6^Clinical Diagnosis and Therapy Center for Glioma of Xiangya Hospital, Central South University, Changsha, China; ^7^Department of Clinical Pharmacology, Xiangya Hospital, Central South University, Changsha, China

**Keywords:** homeobox A5, glioma, cell cycle, cell proliferation, prognosis

## Abstract

Glioma is malignant tumor derives from glial cells in the central nervous system. High-grade glioma shows aggressive growth pattern, and conventional treatments, such as surgical removal and chemo-radiotherapy, archive limitation in the interference of this process. In this work, HOXA5, from the HOX family, was identified as a glioma cell proliferation-associated factor by investigating its feature in the TCGA and CGGA data set. High HOXA5 expression samples contain unfavorable clinical features of glioma, including IDH wild type, un-methylated MGMT status, non-codeletion 1p19q status, malignant molecular subtype. Survival analysis indicates that high HOXA5 expression samples are associated with worse clinical outcome. The CNVs and SNPs profile difference further confirmed the enrichment of glioma aggressive related biomarkers. In the meantime, the activation of DNA damage repair-related pathways and TP53-related pathways is also related to HOXA5 expression. In cell lines, U87MG and U251, by interfering HOXA5 expression significantly inhibit glioma progression and apoptosis, and cell cycle is arrested at the G2/M phase. Collectively, increased HOXA5 expression can promote glioma progression *via* affecting glioma cell proliferation.

## Introduction

Glioma is a malignant tumor that derived from the central nervous system. Four grades were proposed for evaluating its malignancy according to its pathological features, including grades I, II, II, and IV. Grade IV glioma, also called as glioblastoma or glioblastoma multiforme (GBM), is the worst subtype of glioma with median survival time less than 14.6 months (1). High proliferative ability, disordered tumor angiogenesis, and massive tumor necrotic area were recognized as its feature in GBM (2). Recent study proposed different classification of GBM based on its transcriptomic signatures (3, 4). Treatment like the STUPP protocol is able to prolong patient’s survival time but cannot inhibit glioma progression. Therefore, identification of potential target to glioma may assist in improving its prognosis.

HOXA5 belongs to the gene family encoding transcription factors, which contained homeobox. This gene family expressed subsequently and affected tissue developmental and organogenesis (5). HOXA5 is located on chromosome 7 in human and encodes ANTP-class homeodomain protein consisting of 270 amino acids (5). HOXA5 widely participated in the development of organs such as the respiratory system, digestive system, lipid tissue, mammary gland, and so on (5, 6). Therefore, HOXA5 acts a critical role in human development.

Multiple studies reported that HOXA5 was associated with tumor progression, including leukemia, breast cancer, lung cancer, glioblastoma, colorectal cancer, laryngeal squamous cell cancer, and liver cancer (7–9). In breast cancer, HOXA5 affects the expression of TP53 in tumor cells (10), epithelial-mesenchymal transition (11, 12) and apoptosis (13) to affect tumor progression. HOXA5 inhibits non-small lung cancer metastasis through modulating cytoskeletal remodeling (14). HOXA5 also affects tumor cell proliferation and invasion in cervical cancer (15, 16) and lung cancer (17, 18). TP53, a tumor suppressor gene, highly associates with tumorigenesis and cellular response to DNA damage. It was previously reported that HOXA5 can affect tumor progression through regulating TP53 expression (18–20). Therefore, HOXA5 widely affected tumor progression, but its role in glioma is still unknown.

In this work, the expression profile of the HOXA5 in multiple tumor types was mapped. Its association with glioma progression and potential mechanisms were analyzed by using the GO/KEGG enrichment analysis. We identified that HOXA5 as an unfavorable factor for glioma and its expression are highly connected with the activation of p53-related pathways. We also confirmed that HOXA5 affected tumor cells progression through regulating tumor cell proliferation.

## Materials and Methods

### Sample and Data Collection

From the The Cancer Genome Atlas (TCGA) (https://xenabrowser.net/), Chinese Glioma Genome Atlas (CGGA) (http://www.cgga.org.cn/), and Gene Expression Omnibus (GEO) (https://www.ncbi.nlm.nih.gov/) data sets, we collected HOXA5 data from LGG and GBM samples. RNA-seq data for specific tumor anatomic structure in GBM was from Ivy Glioblastoma Atlas Project (http://glioblastoma.alleninstitute.org/).

### Biological Function and Gene Set Enrichment Analyses

Correlation analysis of HOXA5 regarding different pathological features was performed in the TCGA and CGGA data sets with R language (https://www.r-project.org/). Differentially expressed genes between HOXA5 high and HOXA5 low groups with the adjusted p-value <0.05. and the absolute FC larger than 2.0 were considered to be statistically significant. Association between HOXA5 expression and gene sets from the Molecular Signatures Database (MSigDB) were analyzed using gene set enrichment analysis (GSEA). Gene ontology (GO), KEGG (Kyoto Encyclopedia of Genes and Genomes), and HALLMARK analysis was performed using the R package GSVA. Somatic mutations and somatic copy number alternations (CNAs) were downloaded from the TCGA database. Copy number alternations associated with HOXA5 expression were analyzed using GISTIC 2.0.

### Survival Analysis

Patients were subdivided into high and low groups according to HOXA5 expression. The overall survival (OS), progression-free interval (PFI), and disease-specific survival (DSS) rates of patients in low and high groups were compared by the Kaplan–Meier method with log-rank test. ROC was performed to evaluate the prediction performance of HOXA5 expression in various aspects, including 3-year, 5-year OS, and subtype of GBM (classical, mesenchymal, neural, proneural).

### Cell Culture

U87-MG cells and U251 cells, purchased from the Chinese Academy of Sciences, were cultured in DMEM medium with 10% Gibico FBS+1% penicillin-streptomycin, and the medium was changed every 2 to 3 days.

The siRNA of HOXA5, siRNA-112 (sense 5′-3′: GGACUACCAGUUGCAUAAUTT; antisense 5′-3′: AUUAUGCAACUGGUAGUCCTT), siRNA-610 (sense 5′-3′: GCACAUAAGUCAUGACAACTT; antisense 5′-3′: GUUGUCAUGACUUAUGUGCTT) and siRNA-726 (sense 5′-3′: GCAGAAGGAGGAUUGAAAUTT; antisense 5′-3′: AUUUCAAUCCUCCUUCUGCTT) were purchased from HonorGene (Changsha, China). 95 µl serum-free DMEM medium, 5 µl HOXA5 siRNA, and 5 µl Lip2000 were added into the centrifuge tubes in turns. HOXA5 siRNA-112 was discarded because of its low efficiency according to the Western blot assay.

### Western Blotting Assay

HOXA5 primary antibody (Abcam, Cat# ab140636, RRID: AB_2877721) was diluted to 1:1000. β-actin (Proteintech Cat# 66009-1-Ig, RRID: AB_2687938) was diluted to 1:5000 and utilized as the loading control and internal standard. Secondary antibody (Proteintech Cat# SA00001-2, RRID: AB_2722564; Proteintech Cat# SA00001-1, RRID: AB_2722565) was diluted to 1:5000. The total protein concentration was determined using the BCA Protein Assay Kit (Solarbio, China), according to the manufacturer’s instructions. The blots were subjected to three 5-min washes with TBST prior to 1-h incubation with the secondary antibody, repeated washing, and signal development with Western Lightning Plus-ECL.

### CCK8 Assay

The logarithmic growth phase transfected U251-MG and U87-MG GBM cells were obtained and digested for CCK8 assay. 1 × 10^3^ glioma cells and 100 μl of medium were placed into 96-well plates. The absorbance at 450 nm was measured after hatched for 1 h under the condition of 37°C and 5 % CO_2_.

### Colony-Forming Assay

U87-MG cells and U251 cells were digested and plated in six-well plates (300 cells per well) and cultured with 5% CO_2_ at 37°C for 2 weeks. The colonies were then fixed with 4% methanol (1 ml per well) for 15 min and stained with crystal violet for 30 min at room temperature. After photograph, discoloration was performed with 10% acetic acid, and cells were measured absorbance at 550 nm.

### Cell Cycle Assay

Transfected cells were digested, and cell suspension was obtained after centrifuging. Then, cells were washed two to three times with PBS, and we adjusted the number of cells to 1 × 10^6^ cells/ml, 400 µl PBS was added, the cells were gently resuspended to separate as individual cells, 1.2 ml of pre-cooled 100% ethanol was added, and the cells were placed overnight at 4°C for fixation. PI was excited by a 488-nm argon ion laser and was received by a 630-nm pass filter. The percentage of each cell cycle was analyzed using the PI fluorescence histogram.

### Cell Apoptosis Assay

Transfected cells were digested, and cell suspension was obtained after centrifuging. Then, cells were washed two to three times with PBS, 500-µl binding buffer was added, then the cells were gently resuspended to separate into individual cells, followed by staining with 5-µl Annexin V-APC and 5-µl PI solution for 10 min at room temperature and in dark place. The apoptotic cells were measured by the flow cytometer.

### Statistical Analysis

Kaplan-Meier survival curves were generated and compared using the log-rank test. Wilcoxon rank test (nonnormally distributed variables), t test (normally distributed variables), and one-way analysis of variance were used to analyze the expression difference of HOXA5 in different clinical factors, including WHO grades, GBM subtypes, and treatment outcome. The Pearson correlation was applied to evaluate the linear relationship between gene expression levels. Statistical analyses of the colony-forming assay and the CCK8 assay were carried out by GraphPad Prism (version 8.0). The Kolmogorov–Smirnov test was used to assess the normal distribution of data. All tests were two-sided, and P values <0.05 were considered to be statistically significant.

## Results

### HOXA5 Expression Is Elevated in Aggressive Gliomas

**Figure 1:**
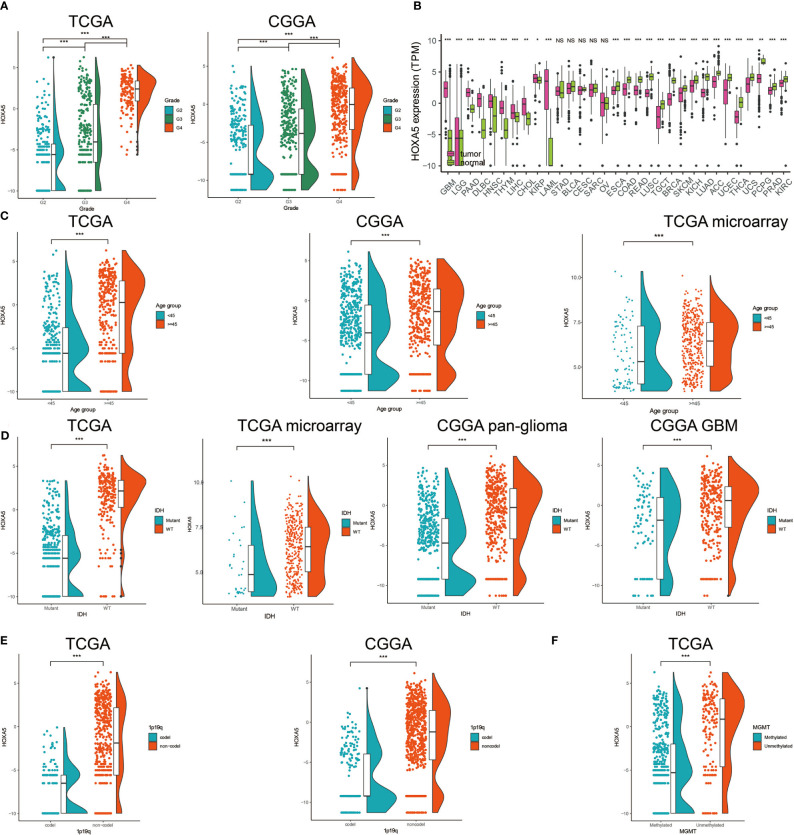
**(A)** Analysis of HOXA5 mRNA levels (log2) in WHO grades II to IV gliomas from TCGA and CGGA. **(B)** Analysis of HOXA5 mRNA levels (log2) in various cancer types. **(C)** The expression level of HOXA5 in different age groups in pan-glioma analysis in TCGA and CGGA data sets, and in GBM patients in TCGA microarray data set. **(D)** HOXA5 was upregulated in the IDH mutant gliomas in TCGA data set, in the IDH mutant GBM in TCGA microarray data set and in both the IDH mutant gliomas and GBM alone in CGGA data set. **(E)** HOXA5 was upregulated in the 1p19q codeletion in pan-gioma analysis in both TCGA and CGGA data sets. **(F)** HOXA5 was upregulated in the methylated gliomas in TCGA data set. NS, Not Statistically Significant; *P < 0.05; **P < 0.01; ***P < 0.001.

The mRNA expression levels of HOXA5 in different WHO grade gliomas were evaluated using expression data from publicly available databases: TCGA, n = 672; CGGA, n = 1013. HOXA5 was observed to be significantly up-regulated in GBM (WHO grade IV) compared with low-grade glioma (LGG) samples (WHO grade III and WHO grade II) in the TCGA and CGGA cohorts (P <0.001, respectively; **Figure 1A**). The expression of HOXA5 was also higher in WHO grade III than WHO grade II cases in the TCGA and CGGA cohorts (P <0.001, respectively; **Figure 1A**). The HOXA5 levels in common cancer types other than gliomas were further evaluated (P <0.001, respectively; **Figure 1B**). We also evaluated the expression levels of HOXA5 in different age groups in pan-glioma analysis in TCGA and CGGA data sets, and in GBM patients in TCGA microarray data set, where patients older than 45 years have higher expression of HOXA5 (P <0.001, respectively; **Figure 1C**). HOXA5 was upregulated in the IDH mutant gliomas in TCGA data set, in the IDH mutant GBM in TCGA microarray data set, and in both the IDH mutant gliomas and GBM alone in CGGA data set (P <0.001, respectively; **Figure 1D**). Furthermore, HOXA5 was upregulated in the 1p19q codeletion in pan-glioma analysis in both TCGA and CGGA data sets (P <0.001, respectively; **Figure 1E**). Additionally, HOXA5 was upregulated in the unmethylated gliomas in TCGA data set (P <0.001; **Figure 1F**).

### Inter-Tumor and Intra-Tumor Heterogeneous Characteristics of HOXA5 in Gliomas

**Figure 2:**
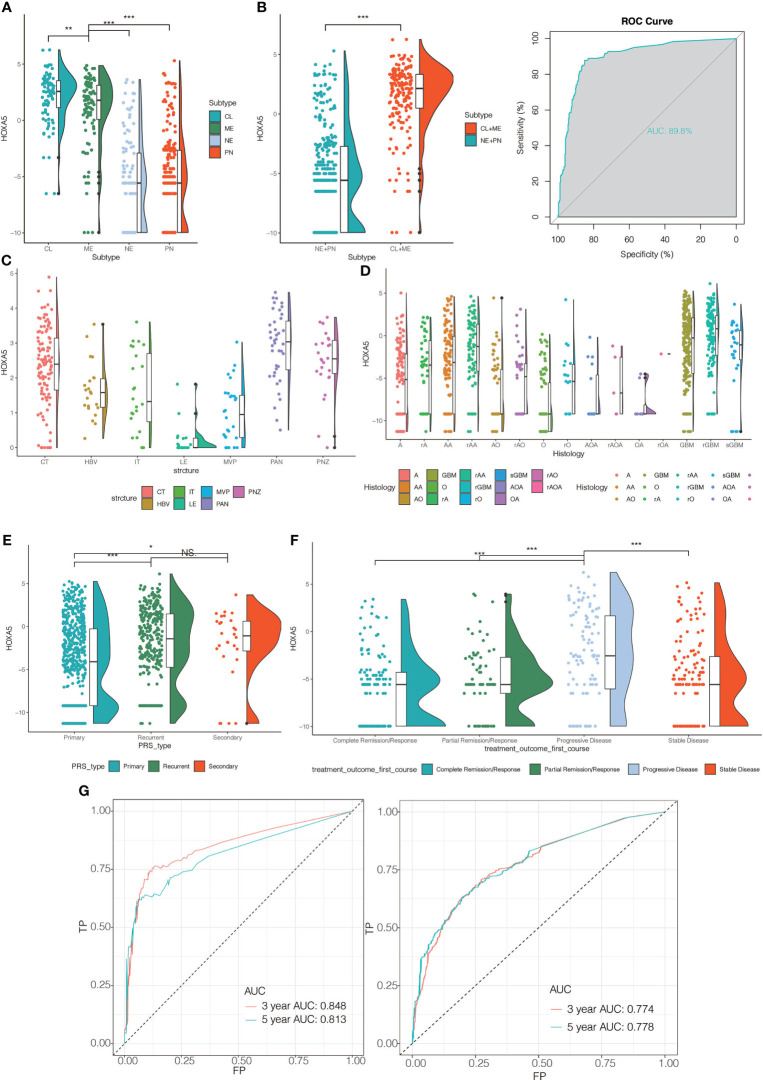
**(A)** The HOXA5 expression pattern in the TCGA molecular subtype in pan-glioma analysis. **(B)** HOXA5 was upregulated in CL and ME subtypes by comparing to NE and PN subtypes in pan-glioma analysis in TCGA data set. Receiver operating characteristic (ROC) curve to assess sensitivity and specificity of HOXA5 expression as a diagnostic biomarker for the ME and CL molecular subtypes in gliomas. **(C)** Intra-tumor analysis of HOXA5 expression using IVY GBM RNA-seq data. Anatomic structures analyzed are the following: LE (leading edge), IT (infiltrating tumor), CT (cellular tumor), PAN (pseudopalisading cells around necrosis), PNZ (perinecrotic zone), MVP (microvascular proliferation), and HBV (hyperplastic blood vessels). **(D)** The expression levels of HOXA5 based on the histopathologic classification. **(E)** The expression level of HOXA5 in primary, recurrent, secondary glioma types. **(F)** The expression level of HOXA5 in different first-course treatment outcomes. **(G)** ROC curve indicating sensitivity and specificity of HOXA5 expression as a diagnostic biomarker for 3- and 5-year survivals in pan-glioma analysis in TCGA and CGGA data sets. A, low-grade astrocytoma; AA, anaplastic astrocytoma; AO, anaplastic oligodendroglioma; GBM, glioblastoma; O, oligodendroglioma; rA, recurrent low-grade astrocytoma; rAA, recurrent anaplastic astrocytoma; rGBM, recurrent glioblastoma; rO, recurrent oligodendroglioma; sGBM, secondary glioblastoma; AOA, anaplastic oligoastrocytoma; OA, oligoastrocytoma. NS, Not Statistically Significant; *P < 0.05; **P < 0.01; ***P < 0.001.

Human gliomas have been molecularly categorized into distinct sub-classes: classical (CL), mesenchymal (MES), proneural (PN), and neural (NE). CL and MES subtypes showed more aggressive growth pattern than PN or NE subtypes (21). We subsequently investigated the inter-tumor heterogeneity of HOXA5 among different molecular subtypes based on the VERHAAK 2010 classification scheme (22). HOXA5 was upregulated in CL and ME subtypes by comparing with NE and PN subtypes in pan-glioma analysis in TCGA data set, where CL subtype had the highest expression of HOXA5 (P <0.001; **Figure 2A**). Receiver operating characteristic (ROC) curve further indicated that HOXA5 might serve as a predictor for CL and MES subtypes in pan-gliomas analysis (AUC value = 0.898, P <0.001; **Figure 2B**). Based on the Ivy Glioblastoma Atlas Project data, HOXA5 was found to be abundant in peri-necrotic zones, pseudopalisading cells around necrosis and cellular tumor compared with other pathological areas (P <0.001; **Figure 2C**). Furthermore, the different expression level of HOXA5 in glioma in regard to histology was shown in **Figure 2D**. HOXA5 was also highly expressed in primary glioma (P <0.001, respectively; **Figure 2E**), whereas the analysis for first-course treatment outcome showed that progressive disease was correlated with higher expression of HOXA5 (P <0.001, respectively; **Figure 2F**).

### HOXA5 Expression Is Associated With Poor Survival in Glioma Patients

**Figure 3:**
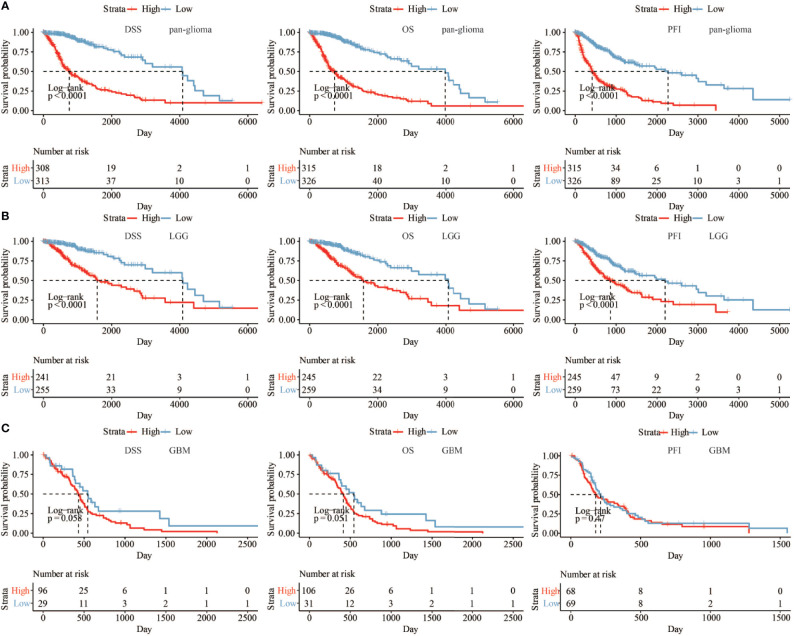
**(A)** The DSS, OS, and PFI survival probabilities of patients in pan-glioma analysis in TCGA data set. **(B)** The DSS, OS, and PFI survival probabilities of LGG patients in TCGA data set. **(C)** The DSS, OS, and PFI survival probabilities of GBM patients in TCGA data set.

**Figure 4:**
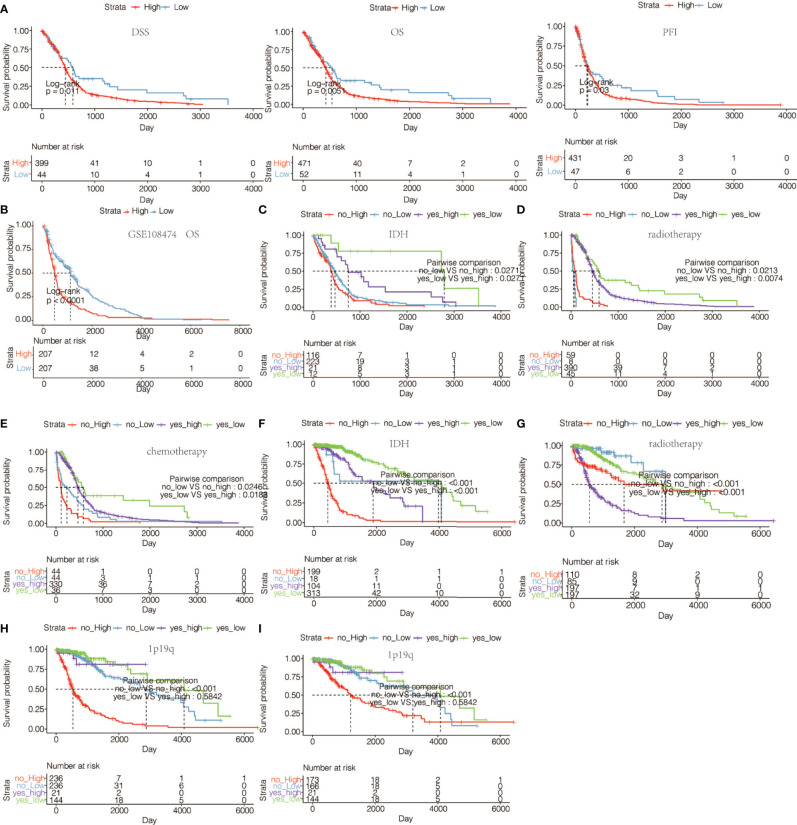
**(A)** The DSS, OS, and PFI survival probabilities of GBM patients in TCGA microarray data set. **(B)** The OS survival probability of glioma patients from GSE108474 data set. **(C)** The OS survival probability of GBM patients without or with IDH mutation with high or low expression of HOXA5 in TCGA microarray data set. **(D)** The OS survival probability of GBM patients without or with radiotherapy with high or low expression of HOXA5 in TCGA microarray data set. **(E)** The OS in GBM patients without or with chemotherapy with high or low expression of HOXA5 in TCGA microarray data set. **(F)** The OS survival probability of patients without or with IDH mutation with high or low expression of HOXA5 in pan-glioma analysis in TCGA data set. **(G)** The OS survival probability of patients without or with radiotherapy with high or low expression of HOXA5 in pan-glioma analysis in TCGA data set. **(H)** The OS survival probability of patients without or with 1p19q codeletion with high or low expression of HOXA5 in pan-glioma analysis in TCGA data set. **(I)** The OS in LGG patients without or with 1p19q codeletion with high or low expression of HOXA5 in TCGA data set.

We next assessed the prognostic value of HOXA5 expression in human gliomas using ROC curve analysis and Kaplan-Meier analysis. The ROC curve analysis showed the prognostic value of HOXA5 in survival in TCGA and CGGA cohorts (3-year AUC value = 0.848, 5-year AUC value = 0.813; 3-year AUC value = 0.774, 5-year AUC value = 0.778, respectively; **Figure 2G**). Kaplan-Meier survival curves were generated based on median values of HOXA5 expression in gliomas. In both TCGA and CGGA data sets, HOXA5^high^ patients exhibited significantly shorter overall survival (OS), disease-specific survival (DSS), progression free interval (PFI) than HOXA5^low^ patients in pan-glioma analysis, (P <0.001, respectively; **Figure 3A**, **Figure S1A**). In addition, HOXA5^high^ patients exhibited significantly shorter OS, DSS, PFI compared with HOXA5^low^ patients in LGG alone in TCGA data set (P <0.001, respectively; **Figure 3B**). Poor OS, DSS, PFI was also associated with high expression of HOXA5 in GBM alone in TCGA data set (P <0.001, respectively; **Figure 3C**). The DSS, OS, and PFI survival probabilities of GBM patients were further testified in the TCGA microarray data set (P <0.001, respectively; **Figure 4A**), in which HOXA5 was correlated with poor survival. Further, HOXA5 predicted worse OS in GSE108474 data set (**Figure 4B**). In TCGA microarray data set, HOXA5 had the highest expression in GBM patients without IDH mutation (P <0.001, respectively; **Figure 4C**), in GBM patients without radiotherapy (P <0.001, respectively; **Figure 4D**), and in GBM patients without chemotherapy (P <0.001, respectively; **Figure 4E**). Similar results were obtained in CGGA data set (P <0.001, respectively; **Figures S1F, S1G, S1H**). In addition, in pan-glioma analysis in TCGA microarray data set, HOXA5 also had the highest expression in patients without IDH mutation (P <0.001, respectively; **Figure 4F**), in patients without radiotherapy (P <0.001, respectively; **Figure 4G**). The results were further confirmed in CGGA data set (P <0.001, respectively; **Figures S1B–D**). Patients without 1p19q codeletion also had the highest expression of HOXA5 in pan-glioma analysis and LGG alone in TCGA microarray data set (P <0.001, respectively; **Figures 4H, I**). Similar results were obtained in pan-glioma analysis and LGG alone in CGGA data set (P <0.001, respectively; **Figures S1E, I**).

### HOXA5 Expression Levels Are Associated With Distinct Genomic Alterations

**Figure 5:**
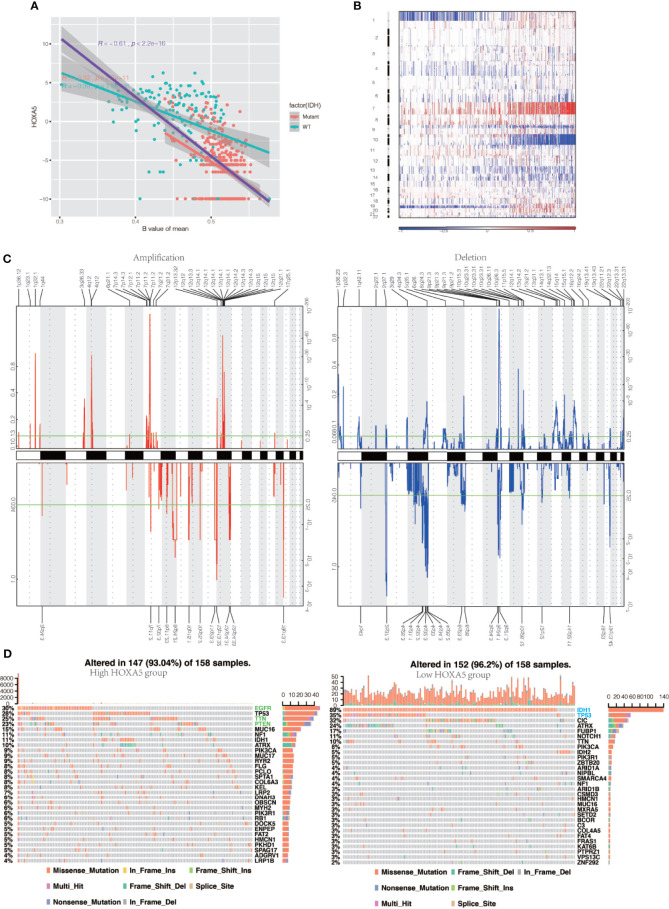
HOXA5 high or low expression is associated with distinct genomic alterations. **(A)** Relationship between HOXA5 and methylation status at promoter region in the cancer genome atlas (TCGA) gliomas samples. The orange dots indicate IDH‐mutant samples, and cyan dots indicate IDH wild‐type samples, respectively. The purple line indicates linear regression between HOXA5 expression and methylation. The orange line and cyan line indicate linear regression between HOXA5 expression and methylation in IDH‐mutant samples and IDH wild‐type samples, respectively. **(B)** Overall copy number variation (CNV) profile according to high *vs* low HOXA5 expression. Blue (deletion); red (amplification). **(C)** Frequency of specific changes based on HOXA5low (lower row) and HOXA5high (upper row) groups. The Y-axis represents the frequency of chromosomal deletion (blue) or amplification (red). **(D)** Spectrum of somatic mutations in gliomas from HOXA5low and HOXA5high groups.

As for the 15 methylation probes designed for HOXA5 from Infinium Human Methylation450 BeadChip, the mean value of methylation probes exhibited negative association with expression of HOXA5 (Pearson test, R = −0.61, P < 2.2 × 10–16). As the IDH mutation exerted great influence on the methylation of the whole genome, we separately analyzed the relationship between HOXA5 and methylation status for IDH‐wild and IDH‐mutant gliomas. As shown in **Figure 5A**, in IDH‐wild gliomas, methylation was much lower than that of IDH‐mutant tumors, as expected (Student’s t test, P = 1.8 × 10^−6^).

To determine whether HOXA5 expression levels were associated with specific genomic characteristics in gliomas, we performed copy number variation (CNV) and somatic mutation analysis using the TCGA data set. A distinct overall CNV profile emerged from the comparison of the HOXA5^low^ (n = 158) *versus* the HOXA5^high^ (n = 158) cluster (**Figures 5B, C**). Amplification of chr7 and deletion of chr10, which are both common genomic events in GBM, frequently occurred in the HOXA5^high^ cluster (**Figure 5B**). Non-deletion of 1p and 19q, a genomic hallmark of oligodendroglioma, also more frequently appeared to be associated with the HOXA5^high^ cluster (**Figure 5C**). In HOXA5^high^ samples, frequently amplified genomic regions included oncogenic driver genes, such as EGFR (7p11.2), IK3C2B (1q32.1), PDGFRA (4q12), and CDK4 (12q14.1), whereas deleted regions contained tumor suppressor genes, including CDKN2A/CDKN2B (9p21.3), PARK7 (1p36.23), and PTEN (10q23.3). In HOXA5^low^ samples, significant amplifications showed peaks in 8q24.21, 12p32.32, and 19p13.3, whereas the frequently deleted genomic regions were 2q37.3, 4q32.3, 9p21.3, and 19q13.43.

Analysis of somatic mutation profiles based on HOXA5 expression levels revealed a high frequency of mutations in EGFR (30%), TP53 (28%), TTN (25%), and PTEN (23%) in the HOXA5^high^ group (n = 158), whereas IDH1 (89%), TP53 (35%), CIC (32%), and ATRX (24%) were more frequently mutated in the HOXA5^low^ group (n = 158; **Figure 5D**).

### Potential Mechanism of HOXA5 in Regulating the Progression of Gliomas

**Figure 6:**
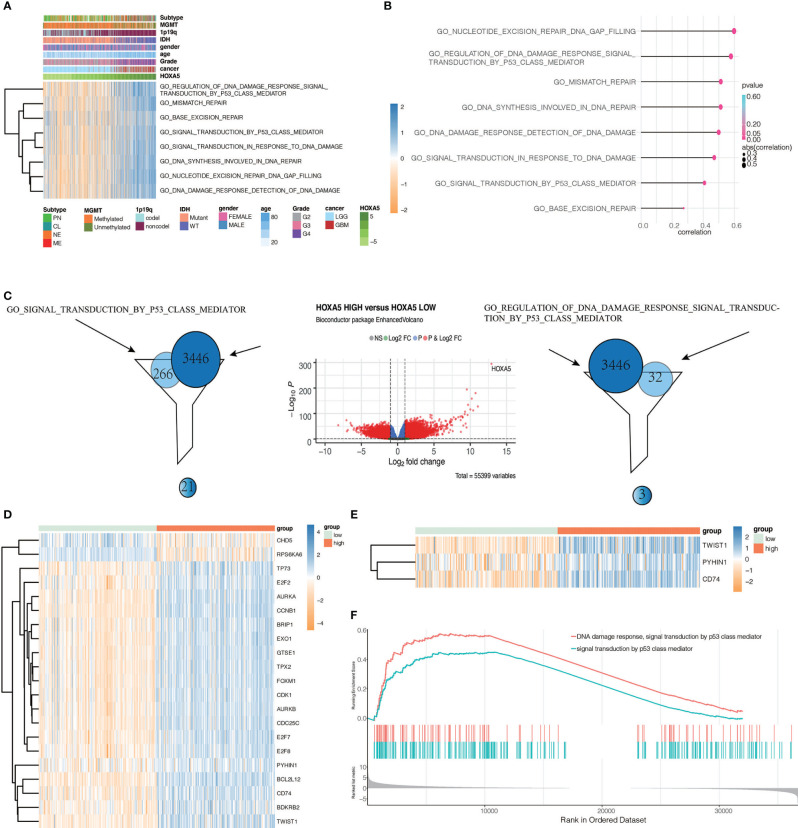
Identifying differentially expressed genes between HOXA5 high and HOXA5 low patients. **(A)** Heatmap of HOXA5-related oncogenic process in pan-glioma analysis in TCGA data set. **(B)** Correlation analysis between HOXA5 and GO pathways. **(C)** Volcano plot of differential gene profiles between HOXA5 high and HOXA5 low groups. **(D)** Genes involved in both regulation of DNA damage response signal transduction by p53 class mediator gene set and DEGs. **(E)** Genes involved in both signal transduction by p53 class mediator gene set and DEGs. **(F)** GSEA plots for enrichment of DNA damage response and p53 signal transduction in HOXA5 high and HOXA5 low samples in the TCGA data set.

**Figure 7:**
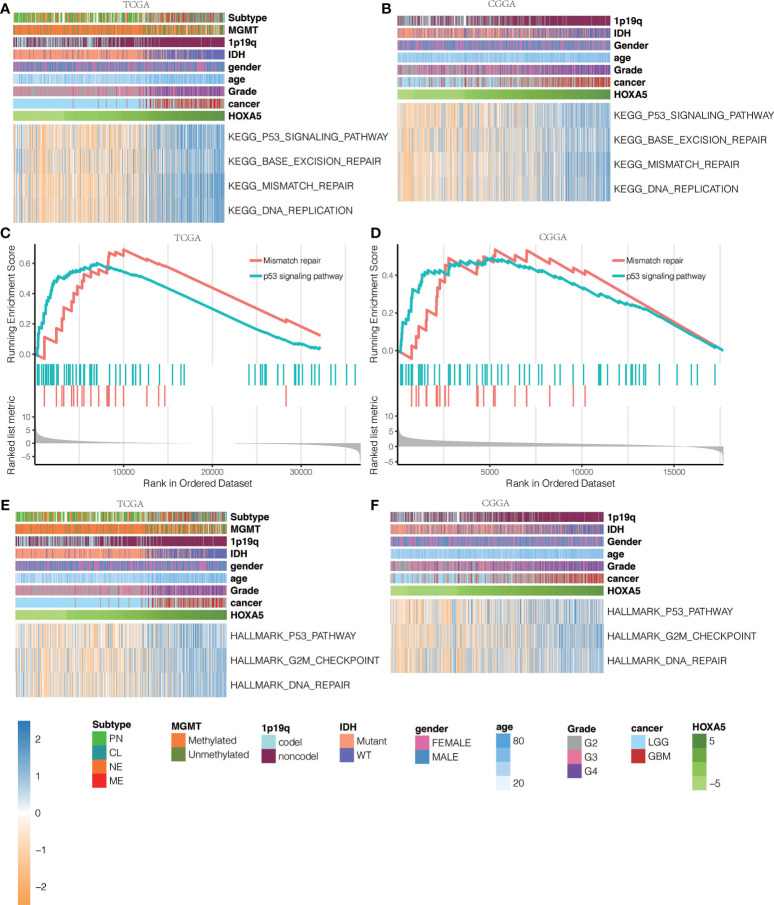
KEGG pathway analysis for mutations based on HOXA5 expression levels in **(A)** TCGA data set and **(B)** CGGA data set. GSEA plots for enrichment of mismatch repair and p53 signaling pathway in HOXA5 high and HOXA5 low samples in **(C)** TCGA data set and **(D)** CGGA data set. HALLMARK pathway analysis for mutations based on HOXA5 expression levels in **(E)** TCGA data set and **(F)** CGGA data set.

To elucidate whether HOXA5 could play a role in promoting gliomas occurrence, GSVA analysis was performed. HOXA5 was found to be associated with regulation of DNA damage response signal transduction by p53 class mediator, signal transduction by p53 class mediator, nucleotide excision repair DNA gap filling, DNA synthesis involved in DNA repair, DNA damage response detection of DNA damage, signal transduction in response to DNA damage, mismatch repair, and base excision repair in TCGA and CGGA data sets (**Figure 6A**, **Figure S2A**). The correlation analysis between HOXA5 and these GO pathways is shown in **Figure 6B**. As the threshold was set as logFC > 2 and adjust P <0.01, a total number of 3,446 differentially expressed genes (DEGs) were detected between high expression of HOXA5 sample and low expression of HOXA5 sample (**Figure 6C**). We paid special attention to two pathways, DNA damage response signal transduction by p53 class mediator and signal transduction by p53 class mediator, in which the GO analysis revealed changes in gene sets related to these two pathways in patients with a higher expression of HOXA5 (**Figures 6D, E**). We further investigated whether HOXA5 might have a role in DNA damage response and p53 signal transduction in gliomas using GSEA analysis in TCGA and CGGA data sets (**Figure 6F**, **Figure S2B**). In KEGG pathway analysis, HOXA5 was found to be associated with mismatch repair, DNA replication, p53 signaling pathway, and base excision repair in TCGA and CGGA data sets (**Figures 7A, B**). The correlation analysis between HOXA5 and KEGG pathways in TCGA and CGGA data sets is shown in **Figure S2C** and **Figure S2D**, respectively. We also investigated the role that HOXA5 might play in mismatch repair and p53 signal transduction in gliomas using GSEA analysis in TCGA and CGGA data sets (**Figures 7C, D**). In HALLMARK pathway analysis, HOXA5 was found to be associated with g2m checkpoint and p53 pathway in TCGA and CGGA data sets (**Figures 7E, F**), in which the correlation analysis is shown in **Figure S2E** and **Figure S2F**, respectively. These results all suggested that HOXA5 was associated with oncogenic processes.

### HOXA5 Affect Glioblastoma Cell Proliferation, Viability, and Apoptosis

**Figure 8:**
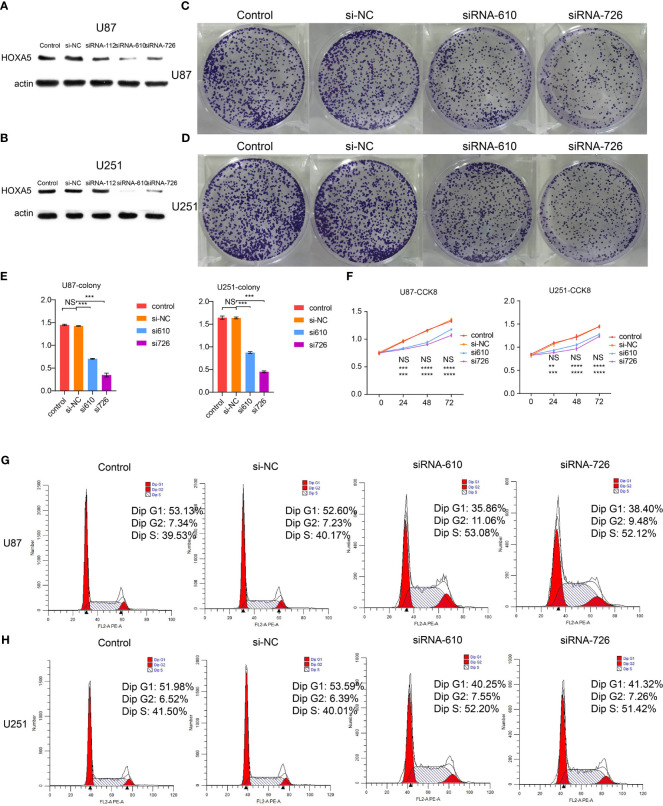
**(A)** The Western blotting results of HOXA5 expression in U87 cell line. **(B)** The Western blotting results of HOXA5 expression in U251 cell line. **(C)** The colony-forming assay, which supports cell viability, is inhibited by silencing HOXA5 expression in U87 cell line. **(D)** The colony-forming assay, which supports cell viability, is inhibited by silencing HOXA5 expression in U251 cell line. **(E)** Statistical analysis for colony-forming assay. NS, not statistically significant; ***P < 0.001. **(F)** Statistical analysis for CCK8 assay. NS, not statistically significant; **P < 0.01; ***P < 0.001; ****P < 0.0001. **(G)** Cell cycle analysis was performed with PI staining by flow cytometry. Representative flow cytometric profiles (left panel) and percentages of cells at the S phase, G1 phase, and G2 phase in U87 cell line are shown. **(H)** Cell cycle analysis was performed with PI staining by flow cytometry. Representative flow cytometric profiles (left panel) and percentages of cells at the S phase, G1 phase, and G2 phase in U251 cell line are shown.

To further investigate the role that HOXA5 plays in the proliferation of GBM, colony-forming assay, CCK8 assay, and cell cycle analyses were performed. Western blot results verified the silence of HOXA5 by siRNA (**Figures 8A, B**). The cell colony forming assay (**Figures 8C, D**) revealed the remarkable suppression of cell clonality of GBM after silencing HOXA5 in U87 cell line and in U251 cell line (**Figure 8E**). The CCK8 assay revealed that the cells proliferation ability is inhibited by silencing HOXA5 (**Figure 8F**). Cell cycle analysis suggested that cells were blocked at the G2/M phase after silencing HOXA5 (**Figures 8G, H**).

**Figure 9:**
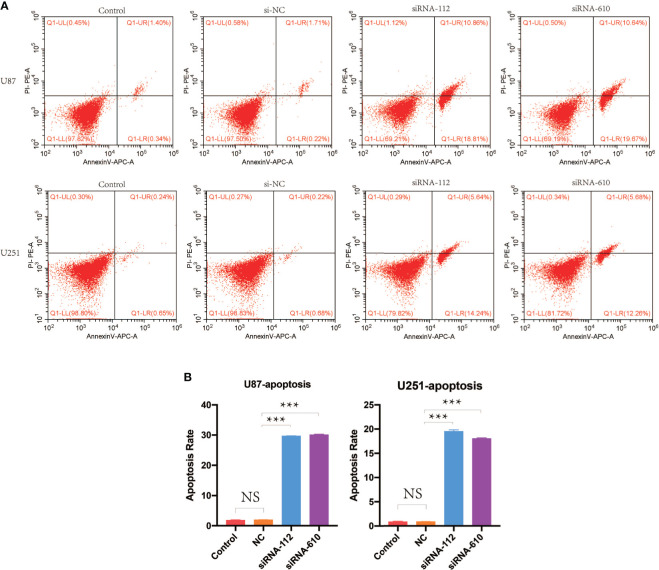
**(A)** Cell apoptosis analysis was performed by flow cytometry. Shown are representative flow cytometric profiles of cells in control group, si-NC group, siRNA-112 group, and siRNA-610 group in U87 and U251 cell lines. **(B)** Statistical analysis for cell apoptosis assay in U87 and U251 cell lines. NS, not statistically significant; ***P < 0.001.

The potential role of HOXA5 in the apoptosis of GBM was also explored. The analysis of flow cytometry described that down-regulation of HOXA5 remarkably enhanced apoptosis in U87 cell line and U251 cell line (**Figures 9A, B**).

## Discussion

Glioma is a central nervous system–derived tumor with highly aggressive growth pattern, treatment resistance, and poor prognosis. Nowadays, strategies show their limitations on improving patient’s survival outcome, including surgery, chemotherapy, and radiotherapy (23). To reveal the mechanism concealed behind its aggressive growth pattern, we identify HOXA5 as prognostic-related genes through regulating cell proliferation. HOXA5, locate on chromosome 7, is involved in tissue development and organogenesis. Multiple studies reported that high HOXA5 expression was a promotor in tumor progression, including esophageal cancer (24), breast cancer (12), gastric cancer (25), and renal cancer (26). In this study, high HOXA5 expression is associated with malignant clinical features like high-grade glioma or IDH wildtype glioma, worse clinical treatment outcome, and strong cells proliferative ability. We also noticed genes like EGFR, PDGFRA were amplified in HOXA5^high^ samples; and genes like CDKN2A/CDKN2B, PARK7 were down-regulated. Those genes were also identified as glioma promotor-related factors (27–33). Besides, tumor cells cycle was arrested at G2/M phase when inhibited HOXA5 expression implying its role in mediating cell cycle. Along with results from other studies that HOXA5 modulated cell cycle in Jurkat cells and hematopoietic stem cells (34), cervical cancer, mesenchymal stem cells (35), and leukemia cells (36). Further, HOXA5 was found to decrease the apoptosis rates of GBM cells. Together, those evidences supported that HOXA5 promoted glioma progression through affecting cell cycle, cell proliferation, and cell apoptosis.

The product of HOXA5 is a DNA-binding transcription factor and able to regulate multiple gene expression, including TP53. Previous study reported that HOXA5 can bind to the promoter of TP53 to activate its transcription and affect corresponded pathways (16, 37). HOXA5 affected tumor progression through influencing TP53 homeostasis in breast cancer (10, 38), TP53-dependent apoptosis in liposarcomas (39), and TP53-mediated cell proliferation in cervical cancer (16). Therefore, HOXA5 can affect TP53-mediated pathway by regulating TP53 expression.

Next, we investigated biofunction difference between HOXA5^high^ and HOXA5^low^ samples. Results suggested that DNA repair-related pathways were activated in HOXA5^high^ samples. Because DNA damage repair is also associated tumor resistance to treatment, such as chemo- or radio-therapy, HOXA5 might also contribute to tumor resistance to treatments (40, 41). In this work, survival analysis based on radio- and chemo-therapy suggested that high HOXA5 samples showed worse clinical outcome than low HOXA5 samples in this study. Besides, previous study confirmed that HOXA5 can affect glioblastoma cells sensitivity to radiation therapy (9). Collectively, HOXA5 may target TP53-mediated DNA damage repair to modulate tumor resistance to treatments (42–44). Taken together, HOXA5 may affect glioma response to chemo- or radio-therapy by targeting TP53.

Other family members in the HOX family are also involved in tumor progression, including leukemia, breast cancer, lung cancer and so on (45, 46). For instance, abnormal expressions of HOXA6, HOXA7, HOXA9, HOXA13, HOXB13, HOXD4, HOXD9, HOXD10, and HOXD13 were noticed in tumor tissue by comparing with normal tissue (47). Those family members can affect glioma cells viability (48, 49), the stemness of glioma stem cells (50), autophagy (51), invasion ability (52), and tumor sensitivity to therapeutic drugs (53). Collectively, this family showed a close association with glioma progression, which deserves further exploration.

In conclusion, this work proved that high HOXA5 expression positively correlated with glioma malignancy’s clinical features and was associated with an unfavorable survival outcome. Moreover, HOXA5 may affect glioma progression and apoptosis by modulating TP53 expression and corresponding pathways. Critically, HOXA5 can arrest cell cycle at G2/M phase to inhibit tumor cell proliferation.

## Data Availability Statement

The original contributions presented in the study are included in the article/**Supplementary Material**. Further inquiries can be directed to the corresponding authors.

## Author Contributions

FD and PC analyzed the data and performed *in vitro* environments. PB assisted in manuscript writing and revising. WP and QC. designed and supervised the study, and offered funding. All authors contributed to the article and approved the submitted version.

## Funding

This study was supported by grants from the research projects Hunan Provincial Natural Science Foundation of China (NO. 2018JJ3838), China Postdoctoral Science Foundation (NO. 2018M633002), Hunan Provincial Health Committee Foundation of China (C2019186). Xiangya Hospital Central South University postdoctoral foundation.

## Conflict of Interest

The authors declare that the research was conducted in the absence of any commercial or financial relationships that could be construed as a potential conflict of interest.

## Publisher’s Note

All claims expressed in this article are solely those of the authors and do not necessarily represent those of their affiliated organizations, or those of the publisher, the editors and the reviewers. Any product that may be evaluated in this article, or claim that may be made by its manufacturer, is not guaranteed or endorsed by the publisher.
