# Supplementary material for: HOXA5 Is Recognized as a Prognostic-Related Biomarker and Promotes Glioma Progression Through Affecting Cell Cycle
Source: Front Oncol. 2021 Aug 17;11:633430. doi: 10.3389/fonc.2021.633430 (PMC8416157; doi:10.3389/fonc.2021.633430)
Supplement: Supplementary file 1 [file DataSheet_1.docx]

Figure Legends:


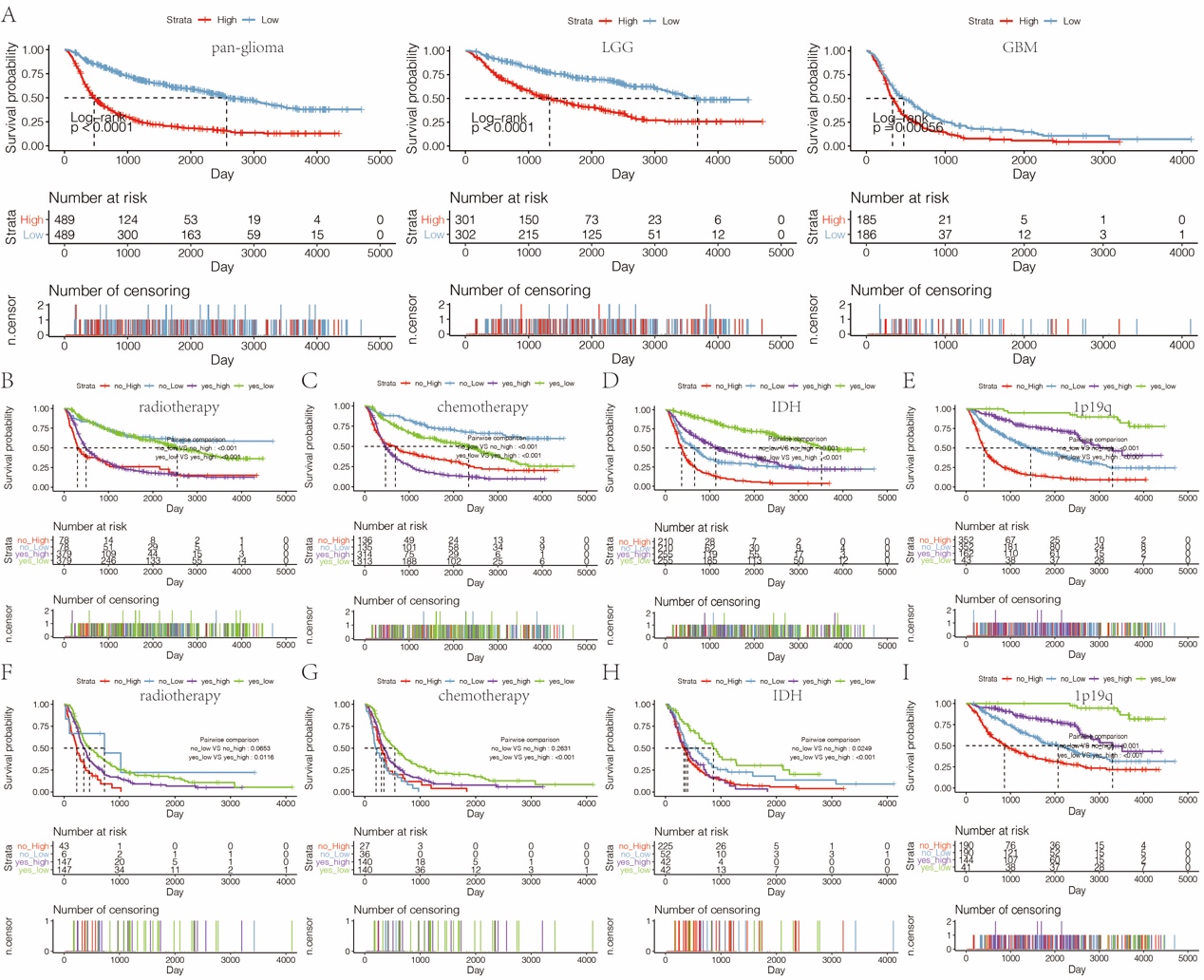


Figure S1. A. The OS survival probability in pan-glioma analysis, LGG and GBM alone in CGGA microarray dataset. B. The OS survival probability of patients without or with radiotherapy with high- or low-expression of HOXA5 in pan-glioma analysis in CGGA dataset. C. The OS survival probability of patients without or with chemotherapy with high- or low-expression of HOXA5 in pan-glioma analysis in CGGA dataset. D. The OS survival probability of patients without or with IDH mutation with high- or low-expression of HOXA5 in pan-glioma analysis in CGGA dataset. E. The OS survival probability of patients without or with 1p19q codeletion with high- or low-expression of HOXA5 in pan-glioma analysis in CGGA dataset. F. The OS survival probability of GBM patients without or with radiotherapy with high- or low-expression of HOXA5 in CGGA dataset. G. The OS survival probability of GBM patients without or with chemotherapy with high- or low-expression of HOXA5 in CGGA dataset. H. The OS survival probability of GBM patients without or with IDH mutation with high- or low-expression of HOXA5 in CGGA dataset. I. The OS survival probability of LGG patients without or with 1p19q codeletion with high- or low-expression of HOXA5 in CGGA dataset.


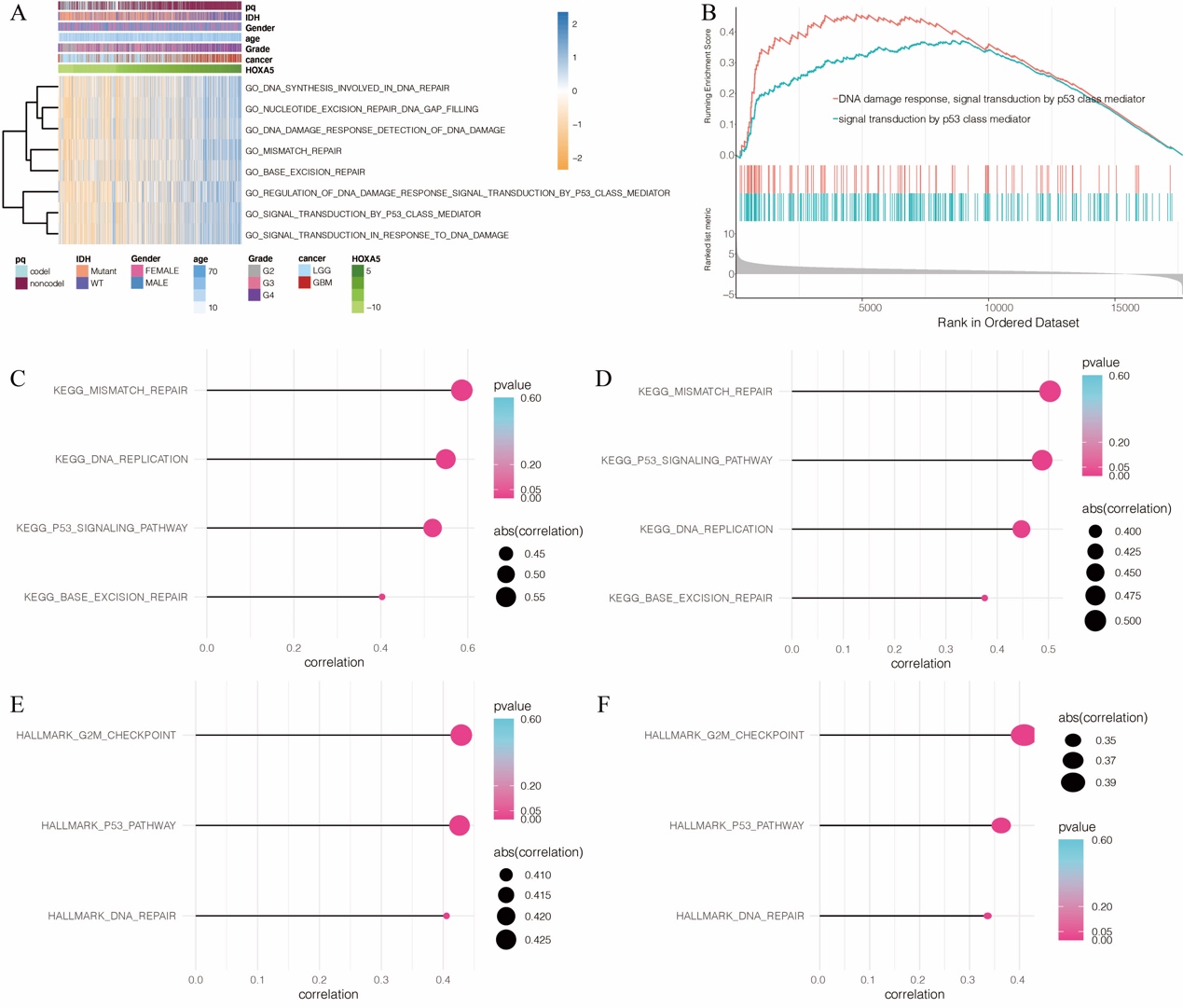


Figure S2. A. Heatmap of HOXA5-related oncogenic process in pan-glioma analysis in CGGA dataset. B. GSEA plots for enrichment of DNA damage response and p53 signal transduction in HOXA5^high^ vs HOXA5^low^ samples in the CGGA dataset. Correlation analysis between HOXA5 and KEGG pathways in C. TCGA dataset and D. CGGA dataset. Correlation analysis between HOXA5 and HALLMARK pathways in E. TCGA dataset and F. CGGA dataset.
